# Interrogating Glioma-Associated Microglia and Macrophage Dynamics Under CSF-1R Therapy with Multitracer In Vivo PET/MRI

**DOI:** 10.2967/jnumed.121.263318

**Published:** 2022-09

**Authors:** Claudia Foray, Cristina Barca, Alexandra Winkeler, Stefan Wagner, Sven Hermann, Michael Schäfers, Oliver M. Grauer, Bastian Zinnhardt, Andreas H. Jacobs

**Affiliations:** 1 European Institute for Molecular Imaging, University of Münster, Münster, Germany;; 2 PET Imaging in Drug Design and Development, Münster, Germany;; 3 Université Paris-Saclay, BioMaps, CEA, CNRS, INSERM, Orsay, France;; 4 Department of Nuclear Medicine, University Hospital Münster, Münster, Germany;; 5 Department of Neurology, University Hospital Münster, Münster, Germany;; 6 Biomarkers and Translational Technologies, Neurosciences and Rare Diseases, Pharma Research and Early Development, F. Hoffmann-La Roche Ltd., Basel, Switzerland; and; 7 Department of Geriatrics with Neurology, Johanniter Hospital, and Centre for Integrated Oncology of the University Hospital Bonn, Bonn, Germany

**Keywords:** glioma, GAMM, ^18^F-DPA-714, TSPO, CSF-1R, C57BL/6

## Abstract

Glioma-associated microglia and macrophages (GAMMs) are key players in creating an immunosuppressive microenvironment. They can be efficiently targeted by inhibiting the colony-stimulating factor 1 receptor (CSF-1R). We applied noninvasive PET/CT and PET/MRI using ^18^F-fluoroethyltyrosine (^18^F-FET) (amino acid metabolism) and *N,N*-diethyl-2-[4-(2-^18^F-fluoroethoxy)phenyl]-5,7-dimethylpyrazolo[1,5-*a*]pyrimidine-3-acetamide (^18^F-DPA-714) (translocator protein) to understand the role of GAMMs in glioma initiation, monitor in vivo therapy-induced GAMM depletion, and observe GAMM repopulation after drug withdrawal. **Methods:** C57BL/6 mice (*n* = 44) orthotopically implanted with syngeneic mouse GL261 glioma cells were treated with different regimens using the CSF-1R inhibitor PLX5622 (6-fluoro-*N*-((5-fluoro-2-methoxypyridin-3-yl)methyl)-5-((5-methyl-1H-pyrrolo[2,3-b]pyridin-3-yl)methyl)pyridin-2-amine) or vehicle, establishing a preconditioning model and a repopulation model, respectively. The mice underwent longitudinal PET/CT and PET/MRI. **Results:** The preconditioning model indicated similar tumor growth based on MRI (44.5% ± 24.8%), ^18^F-FET PET (18.3% ± 11.3%), and ^18^F-DPA-714 PET (16% ± 19.04%) volume dynamics in all groups, suggesting that GAMMs are not involved in glioma initiation. The repopulation model showed significantly reduced ^18^F-DPA-714 uptake (−45.6% ± 18.4%), significantly reduced GAMM infiltration even after repopulation, and a significantly decreased tumor volume (−54.29% ± 8.6%) with repopulation as measured by MRI, supported by a significant reduction in ^18^F-FET uptake (−50.2% ± 5.3%). **Conclusion:** ^18^F-FET and ^18^F-DPA-714 PET/MRI allow noninvasive assessment of glioma growth under various regimens of CSF-1R therapy. CSF-1R–mediated modulation of GAMMs may be of high interest as therapy or cotherapy against glioma.

## Visual Abstract

**Figure:**
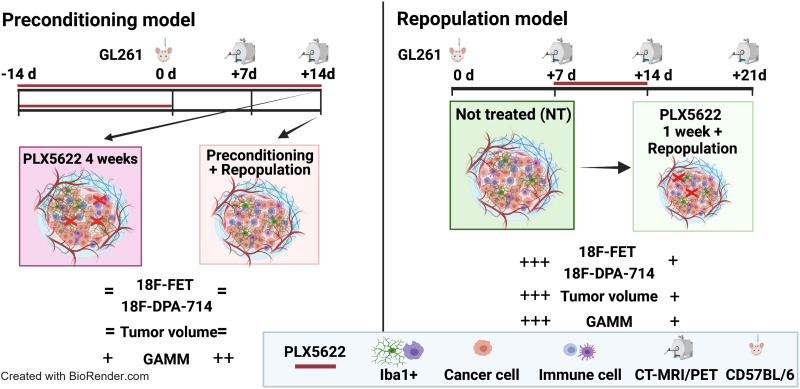

The aggressiveness and molecular complexity of glioblastoma multiforme challenges the current standard-care therapy, limiting the median overall survival to 14–16 mo (*1*). In the last few decades, new immunotherapeutic strategies have been developed for gliomas. Despite some promising results, there are currently no approved immunotherapies that proved to be efficient against glioblastoma multiforme (*2*). The high heterogeneity of the tumor microenvironment plays an important role in therapy resistance, and glioma-associated myeloid cells, such as glioma-associated microglia and macrophages (GAMMs), monocytes, and myeloid-derived suppressor cells (MDSCs) (including polymorphonuclear and monocytic MDSCs), are key players in the establishment of an immunosuppressive environment that favors glioma immune evasion and progression (*3*). Moreover, chronically activated resident immune cells exacerbate the inflammatory response, leading to a high state of neuroinflammation while participating in the development of an immunosuppressive tumor microenvironment (*4*). Therefore, targeting glioma-associated myeloid cells represents an important strategy to develop new glioma microenvironment-targeted therapies. These cell populations are dependent on colony-stimulating factor 1 receptor (CSF-1R) signaling for their survival (*5*). Small-molecule CSF-1R inhibitors are used to study the dynamics of these cells in glioma progression and glioma-associated inflammation, profiting from their ability to modulate GAMMs through a mechanism of depletion and repopulation.

MRI and amino acid PET imaging with methyl-^11^C-methionine or ^18^F-fluoroethyltyrosine (^18^F-FET, amino acid transport) are part of the clinical imaging routine to diagnose and follow up patients with gliomas (*6*–*9*). The use of ^18^F-FET has been reported to have a major clinical value in providing important information for tumor delineation and differentiation and for assessment of posttherapeutic modifications and relapses (*10*). ^18^F-FET PET is highly specific for glioma tissue but falls short in visualizing the reactive and infiltrating myeloid component of the glioma microenvironment (*11*). Other radiotracers, including the 18-kDa translocator protein (TSPO)–targeting PET tracer *N,N*-diethyl-2-[4-(2-^18^F-fluoroethoxy)phenyl]-5,7-dimethylpyrazolo[1,5-*a*]pyrimidine-3-acetamide (^18^F-DPA-714), have been used to visualize the myeloid cell compartment in gliomas (*12*). ^18^F-DPA-714 PET has been reported to give information complementary to that from ^18^F-FET, especially with regard to resident and infiltrating immune cells in preclinical glioma models and in patients (*13**,**14*). Because the timing of immunomodulation may be critical for therapy outcome, noninvasive imaging tools are highly useful in the assessment of the glioma microenvironment before, during, and after therapeutic intervention with novel immunomodulatory compounds (*15*).

Here, we performed a multimodal, dual-tracer imaging study in a syngeneic mouse model of glioma applying ^18^F-FET and ^18^F-DPA-714 PET using a CSF-1R inhibitor to investigate the interplay between glioma progression and related immune cell dynamics. Performing a preconditioning study, we aimed to investigate the possible role of GAMMs in glioma initiation. In the repopulation model, we aimed to assess the therapeutic effect of CSF-1R inhibition–induced depletion and subsequent repopulation on GAMM dynamics in an established tumor.

We hypothesized that a GAMM depletion–repopulation approach would be beneficial, resulting in reduced tumor size and decreased neuroinflammation, and that ^18^F-DPA-714 is a suitable imaging readout for in vivo investigation of GAMM dynamics during the course of CSF-1R inhibitor therapy. Taking advantage of the reversible inhibitory effect of the drug, we were able to noninvasively monitor the dynamics during CSF-1R inhibitor–mediated GAMM depletion and repopulation.

## MATERIALS AND METHODS

### Cell Culture

Mouse GL261 glioma cells were cultured in T-75 cell culture flasks as an adherent monolayer in Dulbecco modified Eagle medium supplemented with GlutaMAX (Thermo Fisher Scientific), 10% heat-inactivated fetal calf serum, and 1% penicillin/streptomycin at 37°C in a humidified incubator maintained at 5% CO_2_ before intracranial implantation.

### Study Approval

All experiments were conducted in accordance with the German law on the care and use of laboratory animals and approved by the Landesamt für Natur, Umwelt, und Verbraucherschutz of North Rhine–Westphalia and the ARRIVE guidelines (Animal Research: Reporting of In Vivo Experiments) (*16*).

### Study Design

**FIGURE 1.**
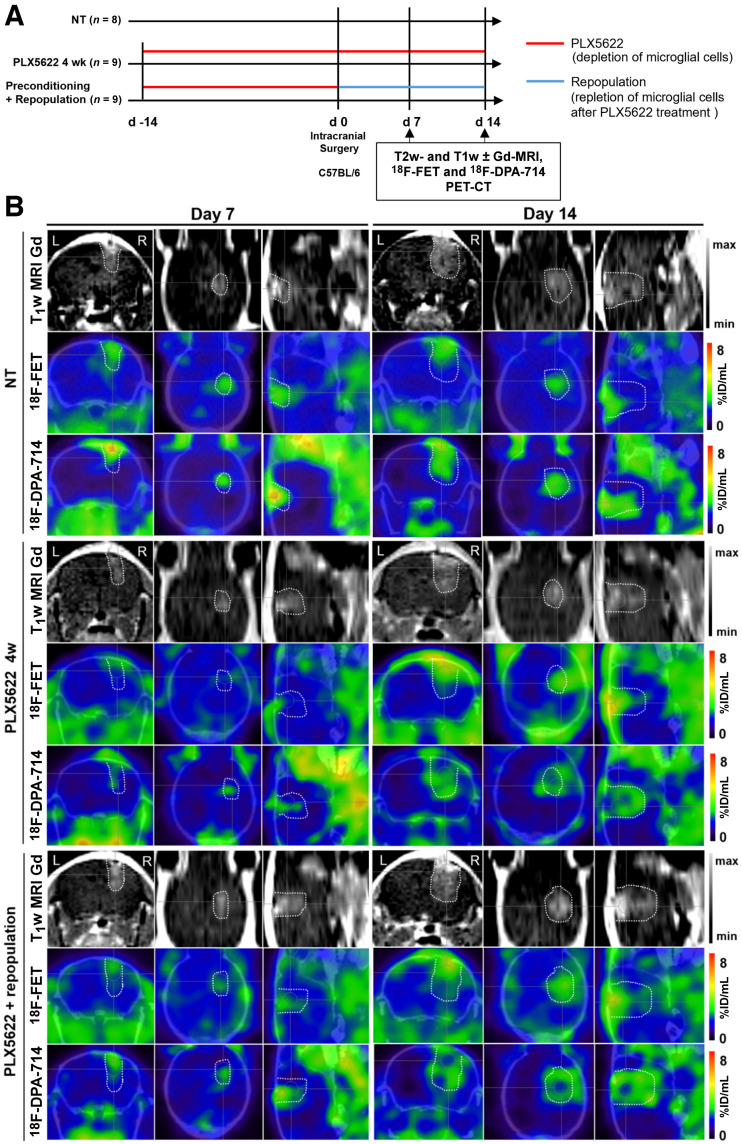
Suitability of longitudinal gadolinium-enhanced T1-weighted MRI, ^18^F-FET, and ^18^F-DPA-714 PET for monitoring immunotherapy-induced changes in preconditioning model. (A) Workflow of preconditioning model. (B) Images during different PLX5622 regimens. Dotted lines indicate tumor area depicted by MRI and transferred to PET images. %ID = percentage injected dose; NT = nontreated.

In total, 51 C57BL/6 female mice 8–10 wk old were orthotopically implanted (intrastriatal injection, coordinates in relation to bregma: lateral, −2.0 mm; anterior–posterior, +0.5 mm; dorsal–ventral, −3.0 mm) with 2 × 10^5^ mouse GL261 cells in 2 μL of NaCl, 0.9%. During all experimental procedures, the mice were anesthetized with 1.5%–2% isoflurane (Abbott Animal Health) in 100% O_2_. After surgery, the animals were weighed daily for at least 3 d and before each imaging session to monitor their health condition (Supplemental Fig. 1; supplemental materials are available at http://jnm.snmjournals.org). Applying different regimens of PLX5622 (6-fluoro-*N*-((5-fluoro-2-methoxypyridin-3-yl)methyl)-5-((5-methyl-1H-pyrrolo[2,3-b]pyridin-3-yl)methyl)pyridin-2-amine), we divided the study into 2 parts, a preconditioning model in which mice were treated with PLX5622 14 d before tumor implantation, and a repopulation model in which microglial cells were acutely depleted and repopulated. The mice underwent sequential gadolinium-enhanced T1-weighted MRI, as well as ^18^F-FET and ^18^F-DPA-714 PET/CT, as described previously (*17*). Full details are available in the supplemental methods and in Figures 1A and 4A.

**FIGURE 2.**
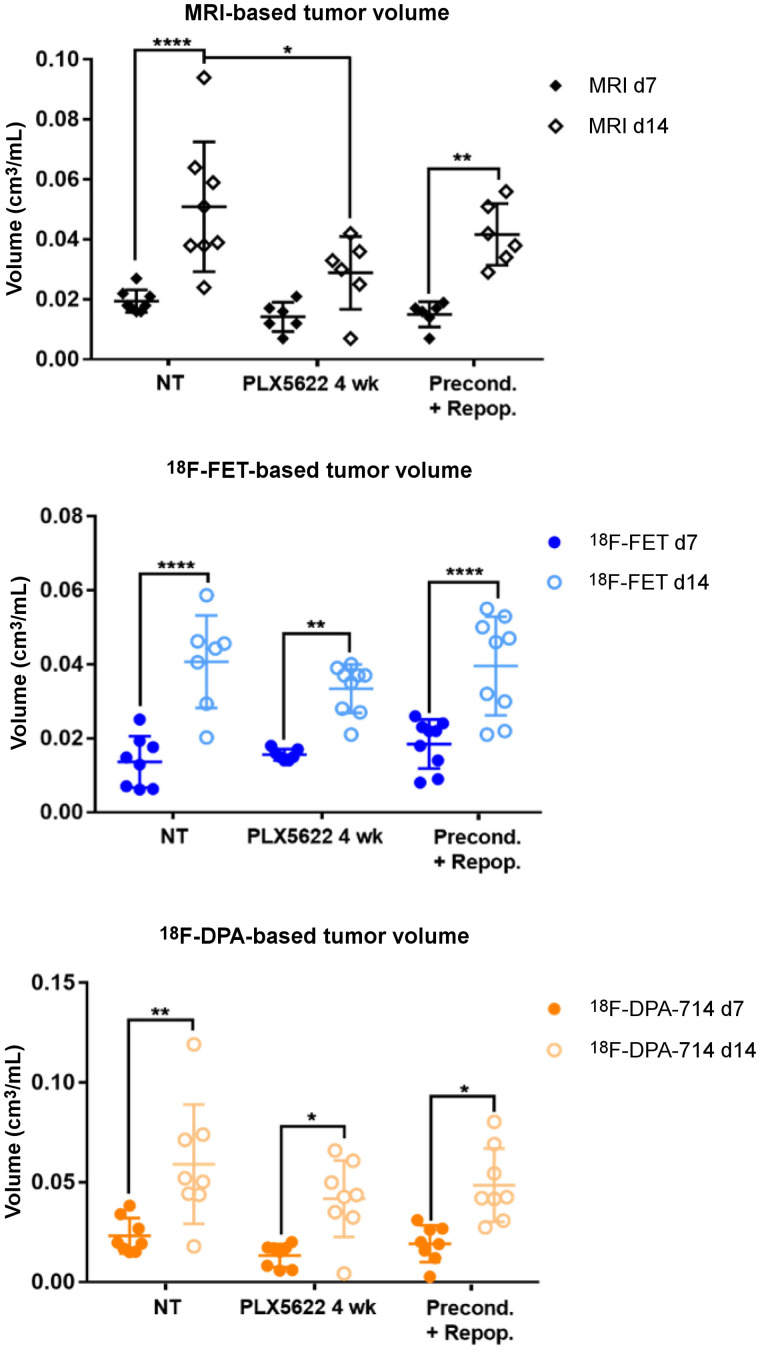
Analysis of tumor and tracer volumes in preconditioning model. Experimental groups include 8 nontreated patients; 9 PLX5622, 4 wk, patients; and 9 preconditioning-plus-repopulation patients. *P ≤ 0.05. **P ≤ 0.01. ****P ≤ 0.0001. NT = nontreated.

### MRI Studies

MRI was conducted for 3-dimensional localization of the glioma and for coregistration with PET/CT images. The mice were anesthetized and the lateral tail vein was cannulated using a 26-gauge catheter. A T1-weighted spin-echo 2-dimensional sequence was acquired in a 1-T nanoScan PET/MRI scanner equipped with an MH20 coil (Mediso Medical Imaging Systems; resolution, 0.27 × 0.27 × 0.9 mm). Gadolinium contrast agent (Gadavist; Bayer Pharmaceutical) was injected via the catheter (50 μmol/kg), and a postgadolinium T1-weighted sequence was acquired.

### PET Studies

PET images were acquired on a high-resolution small-animal PET scanner (32-module quadHIDAC; Oxford Positron Systems Ltd.) with uniform spatial resolution (<1 mm in full width at half maximum). ^18^F-DPA-714 PET images were acquired 60–80 min after intravenous injection of 14.3 ± 2.6 MBq of ^18^F-DPA-714. ^18^F-FET PET images were acquired 20–30 min after intravenous injection of 10.6 ± 0.6 MBq of ^18^F-FET. The supplemental methods provide further details.

### Volumetric Analysis

Imaging data were analyzed using the in-house–developed software MEDgical as described previously (*17*). Briefly, an atlas-based right hemisphere volume of interest was thresholded to delineate tumor and tracer uptake volumes after coregistration of PET/CT scans with MR images. Tumor-to-background ratios were calculated.

### Immunoreactivity

After the last imaging examination, the mice were killed and perfused with 0.9% NaCl and 4% perfluoroalkoxy alkane. The brains were fixed overnight in 4% perfluoroalkoxy alkane, embedded in paraffin, cut into coronal sections, and processed as previously described (*17*). To characterize the tumor microenvironment and therapy-induced modifications, CSF-1R/ionized calcium binding adaptor molecule 1 (Iba1) and TSPO/Iba1 dual labeling with fluorescent antibodies was performed on a preconditioning model and a repopulation model. Positive cells were quantified in biologic triplicates, counting manually or using the bioimage analysis software QuPath (*18*). The antibodies are listed in Supplemental Table 1.

### Multiparametric Flow Cytometry

Myeloid tissue–derived cells were isolated after processing of the tissues as described previously (*17*). The cells were stained with a panel of directly labeled monoclonal antibodies (Supplemental Table 2). All samples were analyzed using the Navios flow cytometer and Kaluza software (version 2.1; Beckman Coulter). The supplemental methods provide additional data.

### Statistical Analysis

Statistical analysis was performed using Prism (version 6; GraphPad Software, Inc.). Differences in radiotracer uptake ratios and tracer uptake volumes between and within groups over time were tested using either 1-way ANOVA with multiple comparisons corrected with a Holm–Šídák test or a *t* test followed by a Mann–Whitney *U* test on ranks and a Wilcoxon test with Bonferroni adjustment for multiple measurements. Correlations between tumor and tracer volumes were tested using Pearson correlation and linear regression. Significance levels were set at a *P* value of less than 0.05. All results are shown as mean ± SE or ± SD. Only animals with full a dataset were considered. Outliers were automatically excluded.

## RESULTS

### ^18^F-DPA-714 PET Imaging Reveals Therapy-Resistant Cells in Chronically Treated Mice

All the experimental groups in the preconditioning study showed similar gadolinium-enhanced T1-weighted MRI and ^18^F-FET–based volume dynamics, together with ^18^F-DPA-714–based volume, indicating increased tumor volume and inflammation over time (Figs. 1B and 2). Tumor volume was significantly reduced in the chronically treated group compared with the nontreated group on day 14. Interestingly, ^18^F-DPA-714 signal was distributed at the borders and in the caudal parts of the tumors, especially in the chronically treated animals, highlighting spatial complementarities to ^18^F-FET uptake (Fig. 1B).

The analyses of the tumor-to-background ratio displayed a significant reduction in ^18^F-DPA-714 uptake already on day 7 in the chronically treated group compared with the nontreated group (Supplemental Fig. 2; Supplemental Tables 3 and 4).

**FIGURE 3.**
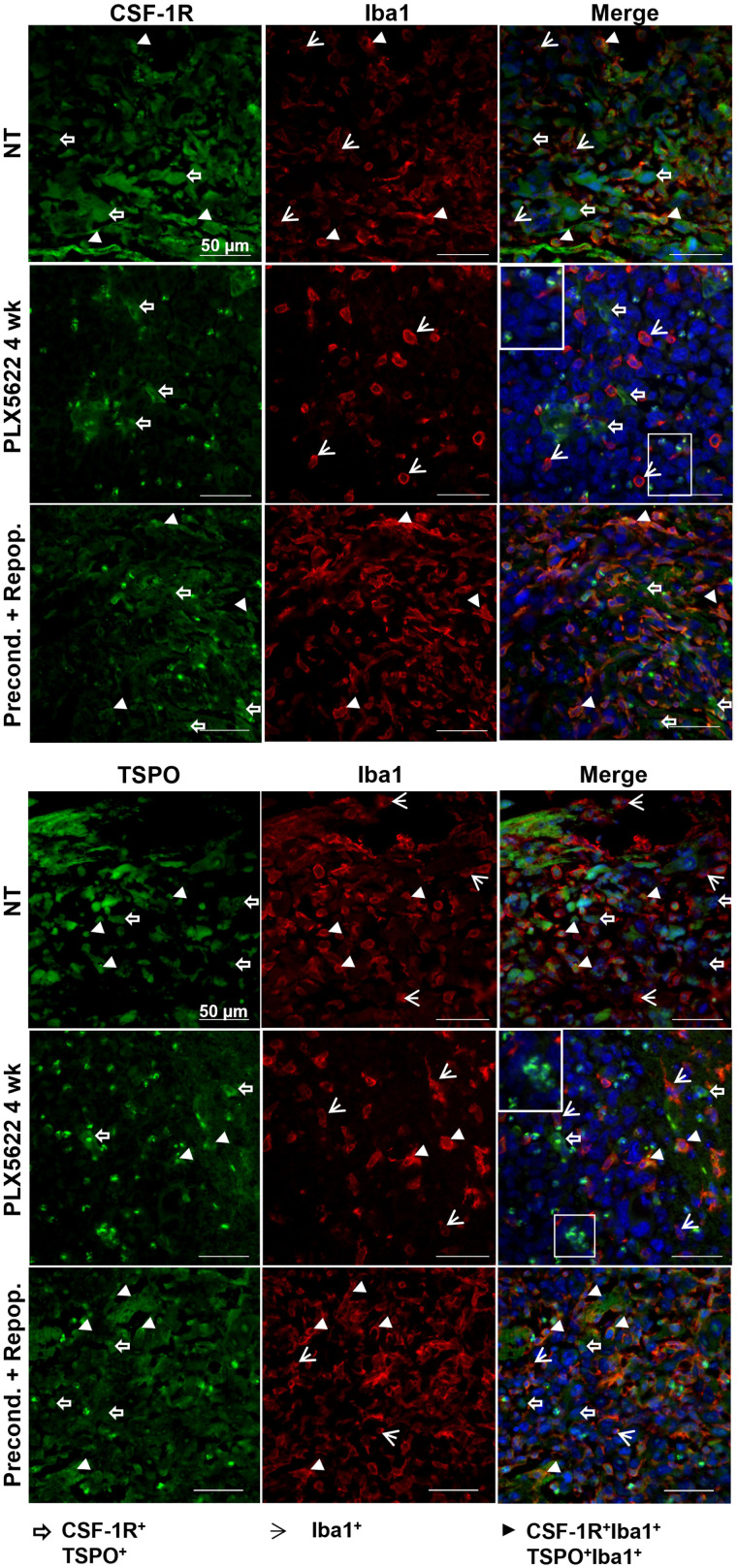
Dual labeling for CSF-1R/Iba1 and TSPO/Iba1 confirms presence of therapy-resistant cell populations. Paraffin-embedded tissues are labeled for CSF-1R/TSPO/Iba1. White boxes show unclassified infiltrating cells. Blue represents nuclear staining with DAPI (*n* = 3 mice/group). NT = nontreated. White open arrows = CSF-1R+ and TSPO+ cells; white arrowheads = Iba1+ cells; white filled arrowheads = CSF-1R+Iba1+ and TSPO+Iba1+ cells.

A high density of Iba1-positive (+) cells was detected in preconditioned-and-repopulated mice, as well as in the nontreated group. Chronic treatment reduced the number of Iba-1+ cells, although not as expected, revealing a resistant Iba1+ cell population in both hemispheres. Repopulation significantly increased the number of Iba1+ cells after preconditioning (Supplemental Fig. 3). Those persistent cells were present within and at the periphery of the glioma and showed reduced CSF-1R and TSPO expression compared with the preconditioned-and-repopulated group; few of the cells expressed both markers. Interestingly, we observed an influx of apparently round CSF-1R+, TSPO+ cells within the tumor area (Fig. 3).

### Volumetric Analyses Show Significant Reduction in Tumor and Tracer Uptake Volumes After Acute Treatment with Subsequent Repopulation

**FIGURE 4.**
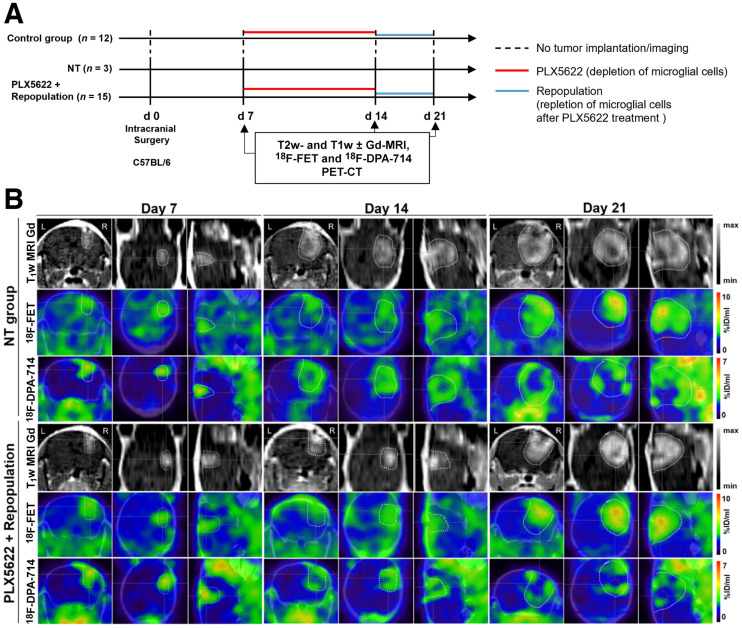
Monitoring glioma immunotherapy-induced changes after GAMM depletion and repopulation using multimodal PET/MRI. (A) Workflow of repopulation model. (B) Gadolinium-enhanced T1-weighted MR and PET images for ^18^F-FET and ^18^F-DPA-714 of nontreated and PLX5622-treated animals, before treatment, after treatment, and after GAMM repopulation (left to right). Dotted line is tumor area depicted by MRI and transferred to PET images. %ID = percentage injected dose; NT = nontreated.

**FIGURE 5.**
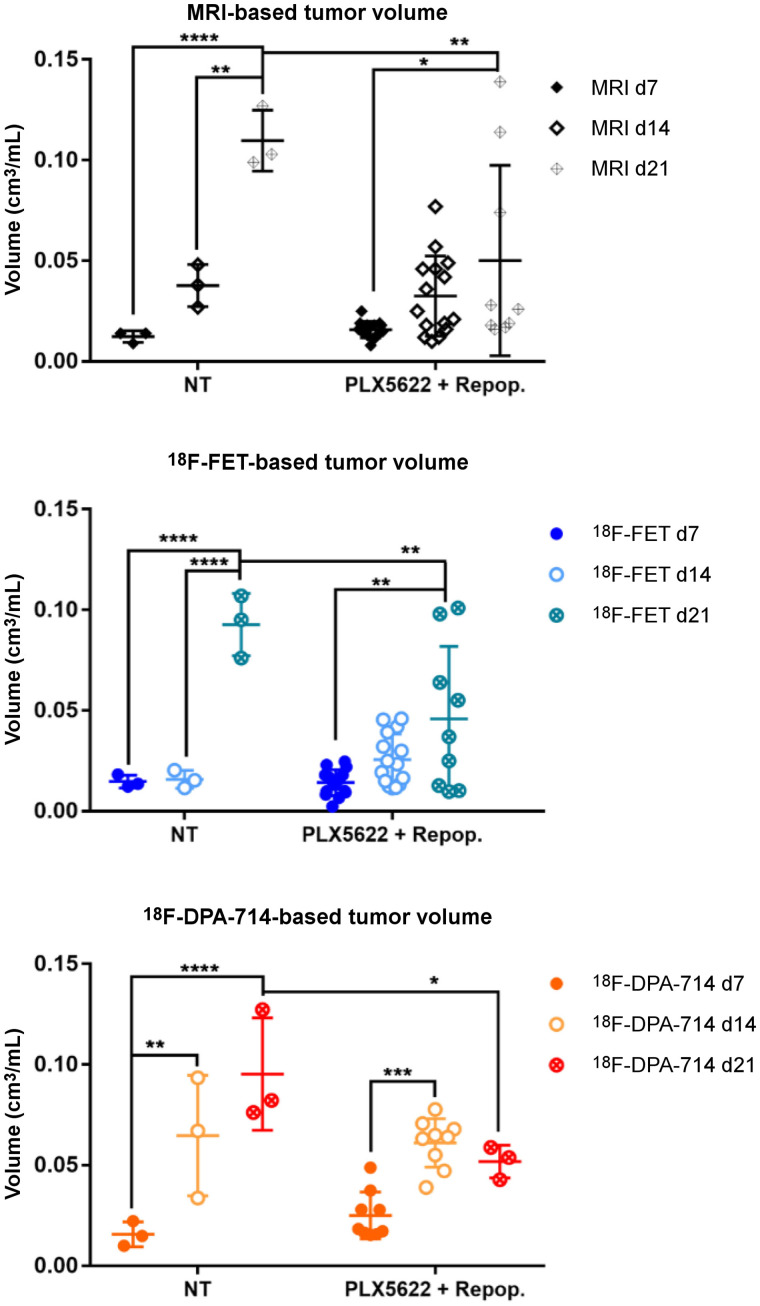
Analysis of tumor and tracer volumes in repopulation model. Experimental groups include 3 nontreated patients and 15 PLX5622-plus-repopulation patients. *P ≤ 0.05. **P ≤ 0.01. ***P ≤ 0.001. ****P ≤ 0.0001. NT = nontreated.

We then assessed the possible effects of acute treatment and subsequent microglial repopulation. In nontreated animals, gadolinium-enhanced T1-weighted MRI, ^18^F-FET PET, and ^18^F-DPA-714 PET showed a significant increase in tumor volume and both tracer volumes over time. In the group treated with PLX5622 (6-fluoro-*N*-((5-fluoro-2-methoxypyridin-3-yl)methyl)-5 -((5-methyl-1H-pyrrolo[2,3-b]pyridin-3-yl)methyl)pyridin-2-amine), gadolinium-enhanced T1-weighted MRI and ^18^F-FET PET–derived volumes significantly increased between days 7 and 21 whereas ^18^F-DPA-714 PET–derived volume significantly increased between days 7 and 14 (Figs. 4B and 5). A therapy effect was observed on day 21, when repopulated animals showed significantly reduced tumor volumes, ^18^F-FET PET–derived volumes, and ^18^F-DPA-714 PET--derived volumes compared with nontreated animals (Supplemental Table 5).

In the nontreated group, ^18^F-FET tumor mean/background mean significantly increased between days 7 and 21, whereas in the PLX5622-plus-repopulation group it increased between days 14 and 21. Similarly, ^18^F-FET tumor maximum/background mean was increased in the nontreated group but was significantly reduced in repopulated animals on day 21 (Supplemental Fig. 4; Supplemental Table 6).

In-depth analyses already showed a statistically significant positive correlation between MRI-based tumor volume and ^18^F-FET uptake on days 14 and 21 (Supplemental Fig. 7).

### GAMMs Are Significantly Affected by CSF-1R Inhibition, with Persistent Reduction in CSF-1R and TSPO Expression After Drug Withdrawal

PLX5622 treatment successfully depleted most Iba1+ cells in glioma-bearing mice compared with the nontreated group (*P* ≤ 0.05), in both the ipsilateral and the contralateral hemispheres, in line with TSPO PET. The remaining Iba1+ cells were at the periphery of the glioma or within the tumor borders, displaying an ameboidlike morphology indicative of an active state. After 1 wk of repopulation, the number of Iba-1+ cells on the contralateral side was significantly increased in the repopulation group compared with the PLX5622 group (*P* ≤ 0.05) (Supplemental Fig. 5).

Similarly, CD68 expression was analyzed as a marker for GAMMs and specifically for macrophage-mediated immune suppression. A large number of CD68+ cells was detected infiltrating the glioma tissue in nontreated animals. The number of CD68+ cells was significantly reduced with PLX5622 treatment and remained significantly reduced after repopulation as shown by quantification (Supplemental Fig. 6).

**FIGURE 6.**
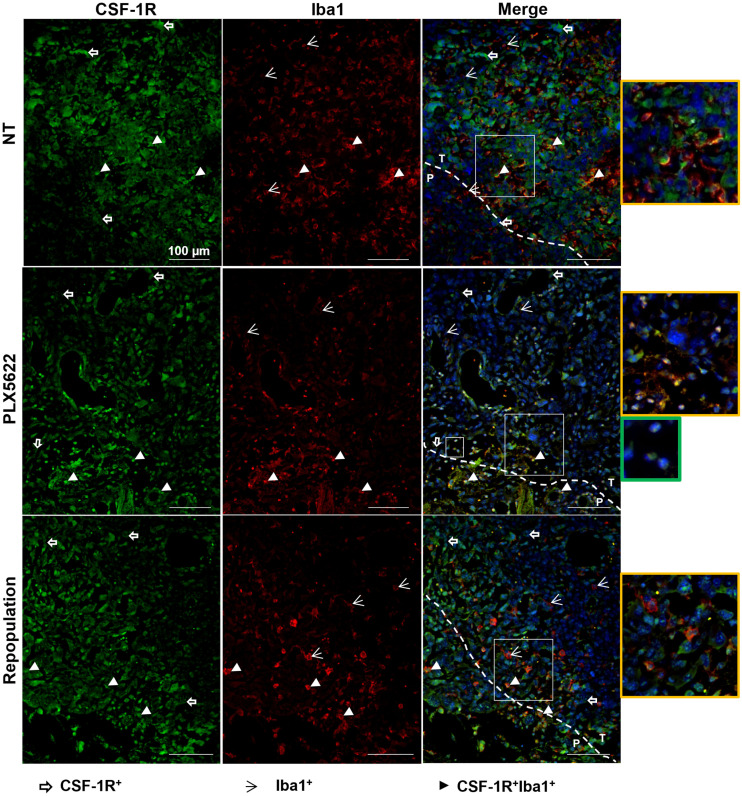
Immunofluorescence labeling for CSF-1R and Iba1 shows persistent reduction in CSF-1R signal after repopulation. Paraffin-embedded tissues are labeled for CSF-1R (green) and Iba1 (red) in nontreated, PLX5622-treated, and repopulated groups. Blue represents nuclear staining with DAPI. Yellow-framed images show magnification of area in larger white boxes. Green-framed image shows magnification of unclassified infiltrating cells (in smaller white box). Dotted line indicates separation between periphery and inner mass (*n* = 3 mice/group). NT = nontreated; P = periphery; T = tumor. White open arrows = CSF-1R+ cells; white arrowheads = Iba1+ cells; white filled arrowheads = CSF-1R+Iba1+ cells.

CSF-1R+ tumor cells, Iba1+ microglial cells at the periphery and infiltrating the glioma, and CSF-1R+, Iba1+ cells within the tumor mass were detected in nontreated animals. PLX5622 treatment reduced CSF-1R expression, with few CSF-1R+, Iba1+ cells present at the border of the glioma. After repopulation, CSF-1R signal remained reduced and CSF-1R+ cells were visible at the periphery of the glioma (Fig. 6).

**FIGURE 7.**
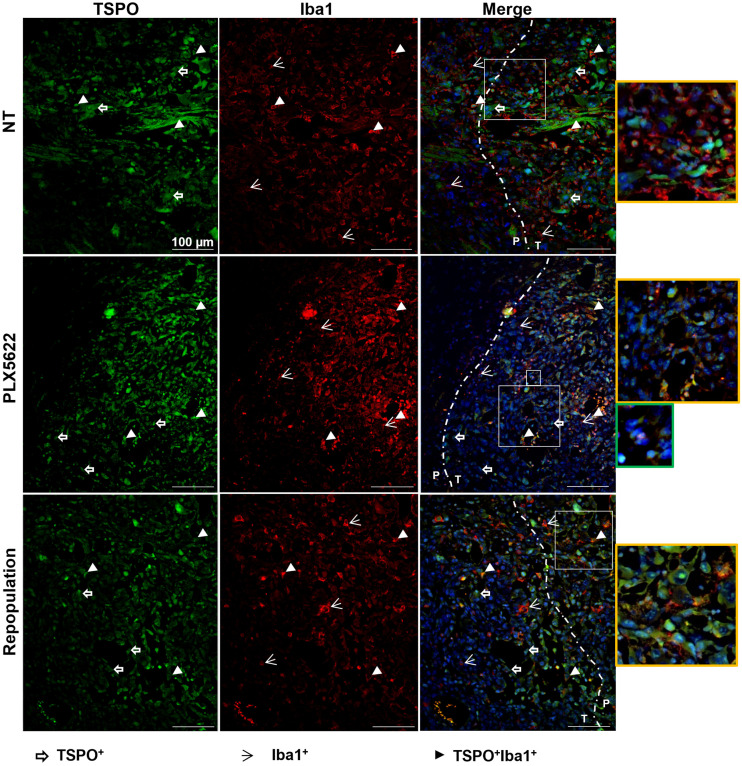
Immunofluorescence labeling for TSPO and Iba1 shows persistent reduction in TSPO signal after repopulation. Paraffin-embedded tissue are labeled for TSPO (green) and Iba1 (red) in nontreated, PLX5622-treated, and repopulated groups. Blue represents nuclear staining with DAPI. Yellow-framed images show magnification of area in larger white boxes. Green-framed image shows magnification of unclassified infiltrating cells (in smaller white box). Dotted line indicates separation between periphery and inner mass (*n* = 3 mice/group). NT = nontreated; P = periphery; T = tumor. White open arrows = TSPO+ cells; white arrowheads = Iba1+ cells; white filled arrowheads = TSPO+Iba1+ cells.

Similarly, TSPO+ cells and Iba1+ cells, as well as TSPO+, Iba1+ cells, surrounded and infiltrated the glioma tissue in the nontreated group. After CSF-1R inhibition, TSPO signal was detectable mostly at the border of the tumor mass, in line with the imaging results, together with a few Iba1+ and TSPO+Iba1+ cells. After repopulation and in accordance with the imaging results, the reduction in TSPO signal remained stable, and TSPO+ cells were visible at the periphery of the glioma whereas cells that were Iba1+, TSPO-negative and Iba1+, TSPO+ were detectable within the glioma tissue (Fig. 7). Interestingly, the depletion of Iba1+ cells seemed to promote the infiltration of as-yet-uncharacterized CSF-1R+, TSPO+ cells (Figs. 6 and 7).

### CSF-1R Inhibition Shows Immune-Modulatory Effects on Glioma-Associated Myeloid Tissue–Derived Cells

To further evaluate therapy-induced changes in the tumor microenvironment and to characterize the phenotype of infiltrating cells, multiparametric flow cytometry was performed (Supplemental Fig. 8). PLX5622 treatment reduced the frequency of CD45+ tumor-infiltrating leukocytes compared with the nontreated group (15.2% vs. 22.3%, respectively), in which the frequency was slightly increased after repopulation (19.1%). The same results were found for GAMMs, confirming the histologic results. Comparable amounts of total MDSCs were detected in the PLX5622-treated and the nontreated groups (CD11b+, Gr1+: 5.1% vs. 3.6%), which significantly decreased after repopulation (CD11b+, Gr1+: 0.78%). Moreover, PLX5622 treatment produced a difference in the MDSC phenotype and affected the expression of different M2 activation markers, such as TSPO and major histocompatibility complex class II. The supplemental appendix provides additional information.

## DISCUSSION

This study aimed to assess the suitability of a multitracer PET/MRI combination to investigate the effects of microglia-depleting immunotherapy in the tumor microenvironment in a syngeneic mouse glioma model. Using different treatment regimens, we demonstrated that the dual-tracer combination of ^18^F-FET and ^18^F-DPA-714 PET in conjunction with MRI allows monitoring of therapy-induced changes in glioma progression and GAMM dynamics in the tumor microenvironment. In contrast to ^18^F-FET PET, ^18^F-DPA-714 PET may be suitable to detect immunotherapy-induced changes within the glioma microenvironment when it is used in conjunction with detailed immunohistochemical analysis. We demonstrated that CSF-1R inhibition affects both glioma cells and specific glioma-associated myeloid-derived cell populations. GAMM inhibition was sustained even after drug withdrawal. Histologic analyses confirmed the efficacy of PLX5622 to deplete most Iba1+ cells over a short period, whereas longer treatment produced a therapy-resistant cell population. Interestingly, the results indicated that microglia cells and GAMMs do not seem essential for glioma initiation. However, a short-term brain repopulation period after CSF-1R therapy is highly effective in slowing glioma progression and maintaining a reduced inflammatory response. Finally, TSPO PET using ^18^F-DPA-714 gives information complementary to that from the imaging marker ^18^F-FET. Altogether, these findings provide new insight into CSF-1R therapy resistance in glioma and identify novel therapeutic glioma targets.

The role of the CSF-1R/CSF-1 axis has been studied in many cancer types, because tumor cells secrete CSF-1 to attract CSF-1R+ myeloid-derived cells to the tumor microenvironment to sustain tumor progression. Therefore, the expression levels of CSF-1R and circulating ligand are regarded as biomarkers for tumor progression, treatment failure, and poor prognosis (*19*). Different CSF-1R inhibitors have been tested in preclinical studies and clinical trials but failed to show substantial efficacy because of acquired resistance to CSF-1R therapy by the glioma microenvironment (*20*).

TSPO is reported to be upregulated by resident (microglia, astrocytes) and peripheral (monocytes, lymphocytes, neutrophils) immune cells during inflammatory conditions (*21*). In glioma tissue, TSPO is expressed by glioma cells, GAMMs, and endothelial cells (*22*). Our group recently described the suitability of TSPO PET using ^18^F-DPA-714 to monitor treatment outcomes in a preclinical glioma model, by identifying areas of myeloid cell infiltration within the glioma microenvironment (*17*).

Our results show the efficacy of CSF-1R inhibition for the blockade of GAMM proliferation and that the combination of ^18^F-FET and ^18^F-DPA-714 PET can be used to monitor CSF-1R therapy–induced changes in the glioma microenvironment. The CSF-1R inhibitor PLX5622 proves to have a disease-changing effect, reducing glioma progression and neuroinflammation, particularly when administered for a short period during established glioma growth.

We report data on ablation of microglia cells and GAMMs before and during glioma development showing that the inhibition of these cell populations before glioma initiation does not influence glioma formation or the inflammatory response. Ex vivo characterization revealed the presence of a potentially resistant Iba1+ cell population as a possible major cellular source of TSPO expression, particularly in chronically treated animals. These findings were supported by in-depth histologic analyses that showed a persistent reduction in TSPO and CSF-1R signals coming from the glioma tissue, whereas a high number of TSPO+ and CSF-1R+ cells were infiltrating the glioma microenvironment. The same holds true for the preconditioned-and-repopulated group characterized by the presence of a high number of GAMMs and microglia cells positive for all the markers infiltrating the glioma tissue. These results might explain the increased ^18^F-DPA-714 uptake over time, highlighting the importance of TSPO PET in detecting glioma-associated cell infiltration.

Whether the observed resistant cell population is pro- or antitumorigenic has yet to be clarified. However, it partially expressed TSPO, supporting the efficacy of TSPO PET in monitoring specific glioma-associated inflammation. Furthermore, TSPO PET has great potential for the characterization and imaging of the glioma-associated immunosuppressive tumor microenvironment, as shown in the first-in-patients study by Zinnhardt et al. (*14*).

The importance of choosing the proper therapeutic window for GAMM modulation is crucial, and TSPO PET may serve this purpose (*23*). In this regard, a short-term (1 wk) CSF-1R inhibition with subsequent brain repopulation during glioma progression led to a significant reduction in tumor volume as quantified by both gadolinium-enhanced MRI and ^18^F-FET PET. In accordance with the literature, CSF-1R inhibition significantly reduced the number of Iba1+ cells in the brain (*24*). However, tumor cells also express TSPO, and the volumetric analysis showed increased ^18^F-DPA-714 PET volumes on day 14 at the same level as in the nontreated group because of glioma progression, which may hide possible immunologically induced changes. Interestingly, after 7 d of brain repopulation, the ^18^F-DPA-714 PET signal remained stable and a significant reduction in tracer volume was detected compared with the control group, suggesting long-lasting treatment effects. These findings were further confirmed by histology. Furthermore, we showed that CSF-1R inhibition significantly reduced the number of GAMMs infiltrating the glioma tissue, identified as CD68+ cells, and the inhibitory effect was detected also after brain repopulation. These results demonstrate that GAMMs are susceptible to acute CSF-1R–mediated intervention even after treatment withdrawal. In line with the results obtained after the chronic treatment, fluorescent labeling revealed the presence of TSPO+ and CSF-1R+ immune cells infiltrating the tumor after depletion of microglia cells. The presence of these potentially therapy-resistant cells supports the importance of microglia in preventing the influx of tumor-associated cells within the tumor microenvironment. Accordingly, previous studies reported increased peripheral immune cell infiltration after microglia depletion (*25**,**26*).

The CSF-1R therapy–induced effects on the immune component were further investigated by multiparametric flow cytometry analyses. Overall, the analyses indicated a strong effect of CSF-1R immune modulation on the MDSC population. The results suggest that the infiltrating component in the absence of Iba1+ cells might be represented by polymorphonuclear MDSCs. Moreover, PLX5622 effects resulted in the modulation of major histocompatibility complex class II and TSPO expression on GAMMs and MDSCs, in line with the reduction in ^18^F-DPA-714 PET volumes. This finding may indicate that repopulation could modulate the immunosuppressive function toward an antitumor phenotype, highlighting the importance of targeting GAMMs.

As reported, single-agent therapy with CSF-1R inhibitors has demonstrated only modest results in glioblastoma clinical trials, showing no significant improvement in the progression-free survival of patients (*27*). Currently, other ongoing studies are combining CSF-1R therapy and immune-checkpoint inhibitors in different types of tumors (*28*). One pilot study evaluating the TSPO PET tracer ^11^C-PBR28 in patients with primary glioblastoma multiforme or melanoma brain metastasis, treated with chemoradiation or immunotherapy, was completed recently; the results are to be determined (NCT02431572) (*29*).

Validation of TSPO PET tracers in clinical settings is necessary to improve the understanding of glioma-associated inflammation and therapy resistance mechanisms.

## CONCLUSION

^18^F-DPA-714 may be a useful imaging biomarker for longitudinal therapy monitoring and assessment of the glioma-associated inflammatory microenvironment, as well as for patient stratification. CSF-1R–targeting intervention and determination of the ideal treatment window for CSF-1R inhibitors may define a promising complementary therapy strategy in glioma.

## DISCLOSURE

This work was supported by the EU Seventh Framework Programme (FP7/2007–2013) under grant 278850 (INMiND); the Horizon2020 Program under grant 675417 (PET3D); the EU/EFPIA/Innovative Medicines Initiative 2 Joint Undertaking (Immune-Image GA831514) under grant 831514; the “Cells-in-Motion” Cluster of Excellence (CiM) Graduate School by the Interdisciplinary Center for Clinical Research (IZKF core unit PIX), Münster, Germany; and the Herbert-Worch-Foundation, Bonn, Germany. No other potential conflict of interest relevant to this article was reported.

## Notes

KEY POINTS **QUESTION:** Is ^18^F-DPA-714 PET suitable to decipher glioma-associated immune cell dynamics and CSF-1R therapy outcomes? **PERTINENT FINDINGS:** ^18^F-DPA-714 PET provided complementary information to ^18^F-FET PET on glioma heterogeneity, allowed the imaging of immune cell dynamics, supported the assessment of brain target engagement, and elucidated CSF-1R inhibition–induced effects in vivo. **IMPLICATIONS FOR PATIENT CARE:** ^18^F-DPA-714 PET represents a promising tool for noninvasive imaging of therapeutic modulations of the immunosuppressive glioma microenvironment.
